# Cognitive biases in clinical decision-making in prehospital critical care; a scoping review

**DOI:** 10.1186/s13049-025-01415-1

**Published:** 2025-06-03

**Authors:** Adam Awanzo, Julian Thompson

**Affiliations:** 1 Helgelandssykehuset Prehospital Clinic, Mo i Rana, Norway; 2 University of Stavanger, Stavanger, Norway; 3 Adult Intensive Care Unit, Southmead Hospital Bristol, Bristol, UK; 4 Severn Major Trauma Network, Bristol, UK; 5 Nord University, Bodø, Norway

**Keywords:** Prehospital, Critical care, Clinical decision-making, Cognitive biases, Scoping review

## Abstract

**Purpose:**

Every day, critical care providers in the prehospital setting respond to time sensitive and outcome-critical emergencies, often in unfamiliar environments with little or no prior knowledge about the patient. In these demanding situations, they must make multifactorial clinical decisions that may be critical for the patient’s life and future health. Errors in this complex decision-making have identified as a significant cause of patient harm and, consequently, there is increasing research focus upon clinical decision-making and risk mitigation in prehospital critical care. Cognitive biases have been identified as a common cause of these systematic errors in the hospital environment and these studies inspired the aim of this article to map current evidence and investigate, “What cognitive biases affects clinical decision-making in prehospital critical care”.

**Materials and methods:**

A scoping review was conducted following Joanna Briggs Institute`s framework, by searching OVID MEDLINE and PubMed, EMBASE, and Cochrane for articles, no restrictions were set for type of article. Articles describing cognitive biases and clinical decision-making in pre-, and in-hospital critical care were included. Additionally, a search in Google scholar was conducted using keywords identified in included articles.

**Results:**

Five hundred unique articles were identified through the search, of which 16 articles examining cognitive biases and clinical decision making in critical care were included, with only two articles focussed exclusively on prehospital critical care. Twenty-eight unique cognitive biases were identified in these articles. The most identified cognitive biases were, anchoring bias, framing effect, availability bias, confirmation bias, overconfidence bias, premature closure, and omission bias. Twelve articles described contributing factors for cognitive biases and these were categorized into 3 main categories. The main categories identified were lack of unbiased feedback, social behaviour and beliefs, and time pressure. Eleven articles proposed mitigation factors, which were categorized into 3 categories, consisting of feedback and follow-up, organizational culture, and education and training.

**Conclusions:**

This scoping review has identified several cognitive biases that affect clinical decision-making, as well as research gaps in both pre- and in-hospital critical care. Identified evidence suggest that both clinicians and organisations are affected by cognitive biases in clinical decision-making in critical care. Future research should aim to establish how these cognitive biases affect clinical decisions in prehospital critical care, and what measures may mitigate the consequent errors, may reduce patient harm, and improve outcomes.

## Introduction

Clinical decision-making in prehospital critical care is complex and challenging. Prehospital critical care providers respond to time-sensitive and outcome-critical emergencies, often in unfamiliar environments, with potential ongoing safety threats to themselves, the patient, and others at scene [1]. They are exposed to expectations and emotions from patients, relatives, and the public, that generate additional personal and professional pressures. In these challenging circumstances, frequently with limited clinical information or history about the patient available, clinicians must make complex multifactorial clinical decisions, that may be outcome critical for the patient’s life and future health. These situations can sometimes be accurately described by Atul Gawande’s statement about clinical decision making that: “the volume and complexity of what we know has exceeded our individual ability to deliver its benefits correctly, safely, or reliably.” [2].

It has been proposed that diagnostic error rates and patient harm in healthcare are unnecessarily high, partially due to the limitations of human cognition and the complexity of the diagnostic process [4]. The World Health Organization (WHO) reports that adverse events are one of the leading causes of death and disability globally [5]. Data from low- and middle-income countries suggests 134 million adverse events occur in hospitals due to unsafe care, resulting in 2.6 million deaths every year [5]. In high-income countries, the WHO estimates that one in 10 patients are harmed while receiving care in hospital and that 50% of these incidents are preventable. The 2017 Annual Report from the Norwegian Directorate of Health identified 4,235 adverse events, with 43.8% of these related to clinical processes. The main causes of adverse events, including severe patient harm and death, are not following guidelines, initiating treatment too late and diagnostic error (delayed or wrong diagnosis) [3], all of which are influenced by errors in clinical decision-making.

Research into complex decision-making is well developed in other disciplines, especially aviation, and behavioural economics. It has also been gaining prominence in hospital healthcare. However, less is known about cognitive biases and clinical decision-making in prehospital critical care, arguably one of the most pressured and outcome-critical phases of care that demands immediate decision-making with incomplete information. If care is to be optimised at this crucial phase, it is important to understand more about the time sensitive clinical decision-making processes in prehospital critical care. Therefore, this scoping review aims to map current evidence and investigate, “What cognitive biases affects clinical decision-making in prehospital critical care”.

## Methods

### Protocol and registration

This scoping review used the PRISMA Extension on scoping reviews [6] and the framework of Joana Briggs Institute (JBI) on scoping reviews [7] with the study protocol published on Open Sciences Framework (OSF) titled: ‘Protocol for a scoping review. Clinical decision-making and cognitive biases.’ [8].

### Eligibility criteria

Only available full-text, English articles describing clinical decision-making and cognitive biases in the pre- or in-hospital critical care environment were included. Critical care is an international used term, however a unified acceptance off its definition is still lacking. This scoping review used the definition of critical care proposed by Kayambankadzanja et al., as “the identification, monitoring, and treatment of patients with critical illness through the initial and sustained support of vital organ functions” [9]. The study population was healthcare professionals. There was no restriction on the field of profession as the aim is to identify research describing cognitive biases affecting healthcare professionals in clinical decision-making in critical care in general and not for just one profession. No restrictions were set for date. Due to the broad and mapping nature of scoping reviews, no restrictions were set for type of article. The results from in-hospital critical care environments were compared with results from prehospital critical care environments and evaluated for transferability.

### Search strategy

A pilot search was conducted to identify relevant search words. These were used to develop the systematic search performed in Medline, Embase, PubMed and the Cochrane Database of Systematic Reviews. The published a priori protocol [8] proposed using the JBI Population, Exposure, and Outcome (PEO) framework [10] but during the literature search it became evident that a Population, Concept, Context (PCC) framework was more suitable: Population defined as healthcare professionals, Concept being cognitive bias in the Context of critical care. The search strategy was developed and revised in collaboration with a librarian from NORD university. References of included articles were screened for relevant articles and a search in Google scholar using keywords was performed to map evidence which might have been missed from the structured database search. In addition, a hand search for grey literature were conducted using keywords from the structured search for eligible articles. For the Google scholar search a predefined cut off for articles that would be screened by title and abstract was set to the first 100 articles.

### Study/Source of evidence selection

**Fig. 1:**
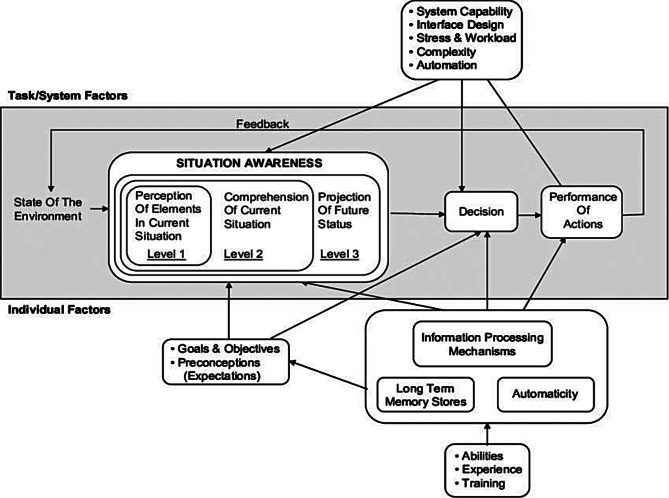
Endsley’s situation awareness model [11]

Records were screened by title and abstract, and index terms used to describe the article. After screening of title and abstract, a second search was performed, based on relevant identified index terms and keywords in all included databases. Relevant articles from the reference list of identified articles were also evaluated. Articles were uploaded to RAYYAN, and duplicates were removed. Initial screening was performed non-blinded independently by both authors, before articles were assessed in full text by the first author. Disagreements during study selection and data extraction were resolved through discussion between the two reviewers. Included and excluded articles were recorded with the main reason for exclusion and are available upon reasonable request to the corresponding author. A piloting of data extraction points was conducted, although the extraction was not stated in the a priori protocol [8] due to the mapping nature of this review. To answer the question of what cognitive biases affect clinical decisions in prehospital critical care, a mapping of the evidence was conducted, extracting data on cognitive biases, contributing factors and mitigating factors. For data analysis this scoping review utilized several methods. During this process no framework or categorization of cognitive biases were found in the literature. Therefore, this scoping review utilizes a categorization according to Endsley`s model for situation awareness [11] (Fig. 1).

For the analysis of cognitive biases, a descriptive analysis and a deductive basic qualitative content analysis were utilized, using Endsley´s model for situational awareness as a coding matrix to categorize the biases according to the levels which they may influence. The analysis of contributing factors and mitigation factors follows an inductive approach, allowing the categories to emerge from the data through manifest coding. This qualitative content analysis is guided by a post-positivism paradigm following Elo and Kyngäs as recommended by JBI [12].

## Results

**Fig. 2:**
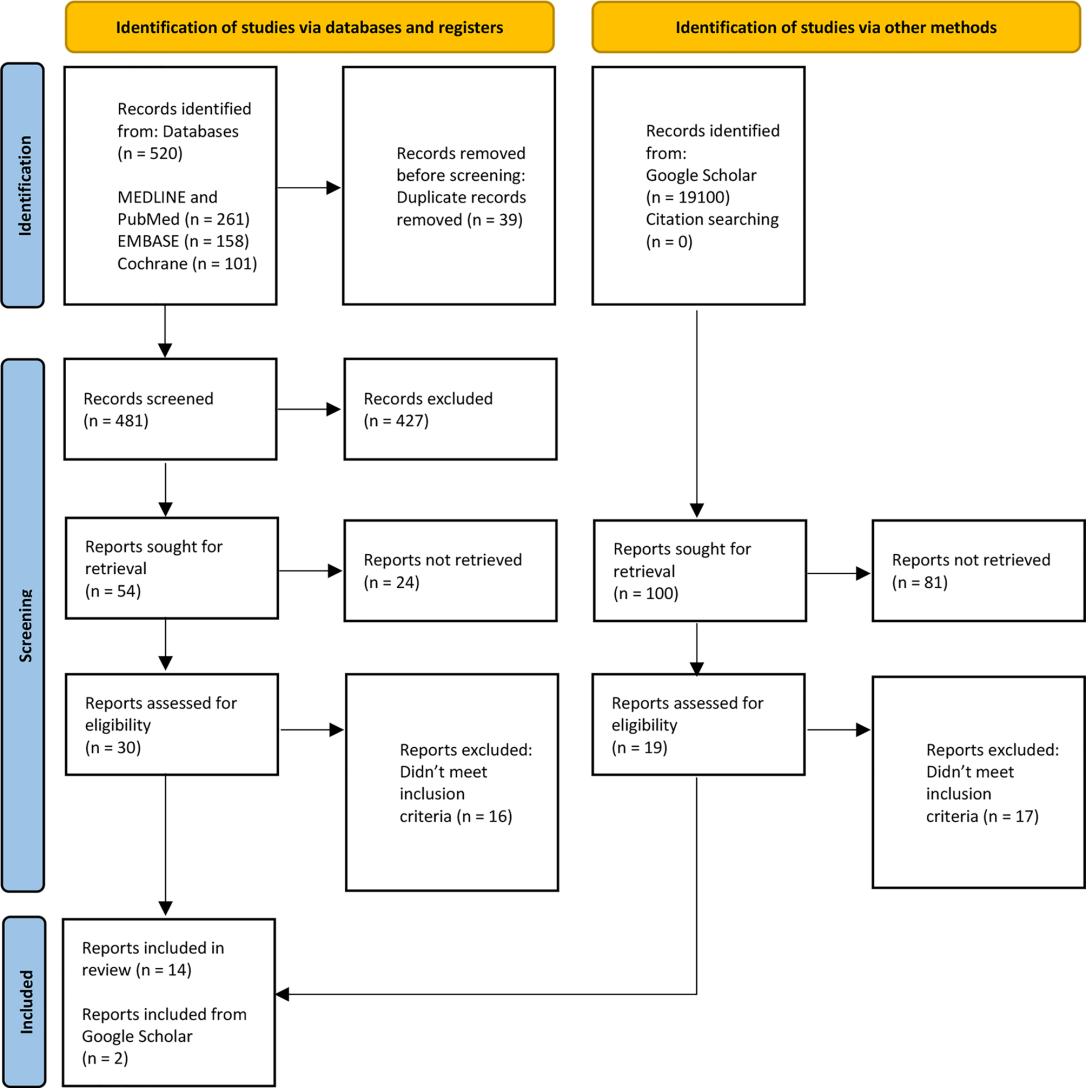
PRISMA Flow diagram. From: Page MJ, McKenzie JE, Bossuyt PM, Boutron I, Hoffmann TC, Mulrow CD, et al. The PRISMA 2020 statement: an updated guideline for reporting systematic reviews. BMJ 2021;372:n71. doi: 10.1136/bmj.n71 [13]

Study characteristics extracted are presented in Tables 1 and 2. 16 articles from 5 countries, published from 1993 to 2023, describing 28 unique cognitive biases were included in this scoping review. Most articles described in-hospital critical care, only two of the included articles describe the prehospital critical care. Of the 16 included articles, 9 were primary research. Of those 6 of 9 (67%) were quantitative. Most articles included in this scoping review were published in the US (56%) (Fig. 2).

**Table 1:** Source of evidence and country of origin

	USA	UK	Australia	Austria	Netherland	Percentage of total by method
Quantitative	5	1				37,50%
Discussion article	2	1	1			25.00%
Evidence synthesis	1	1			1	18,75%
Qualitative	1	1				12,50%
Mixed methods				1		6,25%
Of total by country	56,25%	25,00%	6,25%	6,25%	6,25%	100%

**Table 2:** Overview of extracted data for analysis

Author(s)	Year of publication	Origin/country	Clinical environment	Decision analysed	Patients	Biases	Factors identified	Mitigation	Provider	Primary Research	Evidence synthesis	Discussion article
Aberegg, S et al.	2020	USA	x	x		x	x	x	x	x		
Beldhuis, I et al.	1993	USA	x			x		x			x	
Christakis, N and Asch, D	2021	UK	x	x	x	x			x	x		
Eichinger, M et al.	2021	Netherland	x		x	x			x		x	
Fassier, T et al.	2019	Austria	x	x	x	x		x	x	x		
Fiadjoe, J and Nishisaki, A	2022	USA	x	x	x	x		x				x
Freshwater- Turner, D.A et al.	2005	USA	x	x	x	x		x	x			x
Hayes, Margaret M et al.	2021	UK				x		x				x
Lighthall, G and Vazquez- Guillamet, C	2015	USA	x			x	x	x			x	
Lucas, N et al.	2021	USA	x	x		x	x	x		x		
Marsden, M et al.	2015	USA	x	x	x	x	x		x	x		
Mehta, H and Plunkett, A	2012	UK				x		x				x
Ordoobadi, A et al.	2019	USA	x	x	x	x	x	x	x	x		
Stanak, M and Hawlik, K	2007	Australia	x	x	x	x	x	x	x	x		
Steinberg, A et al.	2017	USA	x	x	x	x	x		x	x		
Yang, H et al.	2023	UK	x	x		x	x		x	x		
Number of articles presenting			14	11	9	16	8	11	10	9	3	4
Percentage presenting of total			88%	69%	56%	100%	50%	69%	63%	56%	19%	25%

### Clinical environment

Of the 14 studies reporting on specific clinical environments, 4 articles described both ICU and ED [17, 20, 26, 28]. Three articles described ICU only [16, 26, 31]. Three articles described prehospital care [15, 27, 32]. One reported on Post-Cardiac Arrest Service/ICU [22]. Another reported on neonatal ICU [19] and one reported on OR, ICU and ED [30].

### Cognitive biases

**Fig. 3:**
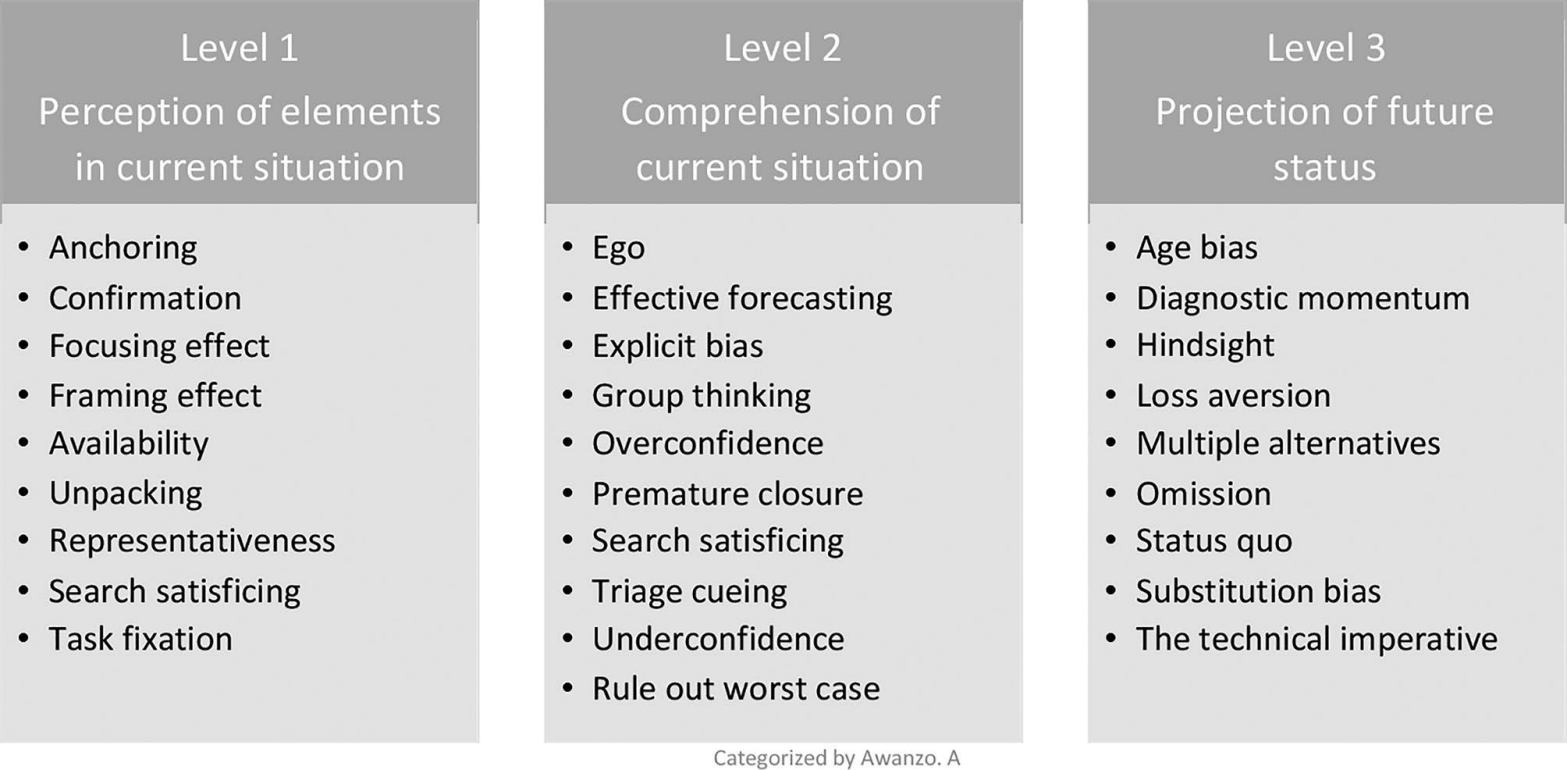
Identified cognitive biases categorized by level of situation awareness from Endsley`s model [11]

Most articles reported 2 or more cognitive biases that influence decision-making in critical care. Three articles reported on a single bias and of these, 2 studies reported on age bias, and one reported on overconfidence. Of the 28 unique cognitive biases identified, 11 were mentioned in 2 or more articles. Nine were reported and described in 2 or more articles. These are presented categorized by the levels of Endsley`s model for situation awareness (Fig. 3).

### Perception of elements in current situation

#### Anchoring bias

Anchoring occurs when a clinician focuses too early and heavily on some specific initial findings in the diagnostic process. For example, if an initial clinical presentation of an aortic dissection is assessed by a clinician as being a likely myocardial infarction, then instead of broadly evaluating all the available information, there may be an inability to utilise conflicting data to adjust from a presumptive diagnosis of myocardial infarction to aortic dissection [14].

Three studies reported on Anchoring bias [15–17]. Marsden et al., performed semi structured individual interviews of experienced prehospital consultants, exploring decision-making for pre-hospital blood transfusion in trauma patients. They discovered examples during interviews highlighting that participants showed overdependence on initial information during clinical decision-making, thereby presenting the presence of anchoring bias [15]. A registry study from Lucas et al., of patients admitted to a noncritical care level after being evaluated in the ED and then upgraded to ICU within 48 h, found that for the year 2019, anchoring bias was the most prominent cognitive bias in non-concordant diagnosis [16]. Fassier et al., carried out a qualitative study of physicians who are making end-of-life decisions for elderly patients, they found that physicians relied prematurely and strongly on prominent presentation features [17].

#### Framing effect

Framing effect is described as the tendency to base judgements based on how the information is presented. For example, the information in patient handover will potentially frame how the receiving team evaluates and makes decisions. Tversky and Kahneman provides a different example: *“The odds of survival one month after surgery are 90%” is more reassuring than the equivalent statement that “mortality within one month of surgery is 10%.”* [18].

Three studies reported on framing effect [15, 17, 19]. During their interviews Marsden et al., found that examples of framing effect were evident in 5 of the 10 interviews conducted [15]. In the study from Fassier et al., they observed that framing effect influenced clinical decision-making, but did not highlight it as one of three main influencing biases [17]. A study from Stanak et al., looking at decision-making at the limit of viability in neonatal ICU discussed how framing effect influences shared decision-making. They state that how information is presented, and the order of information may affect parents and clinicians [19].

#### Availability bias

Availability bias is described as trusting or relying on the most easily available information. Tversky and Kahneman describes it as *“judging frequency by the ease with which instances come to mind”.* They describe that dramatic events or personal experience increases availability of such events [18]. For example, a physician who frequently responds to myocardial infarctions who is presented with an aortic dissection might be more likely to trust easily available information that this resembles an myocardial infarction. On the contrary if the same physician has experienced a dramatic episode with misdiagnosis of an aortic dissection the opposite is likely to occur under the influence of availability bias [20].

Two studies reported on Availability bias [15, 16]. In the study of Marsden et al., prehospital physicians articulated the tendency to trust information that easily comes to mind, here by demonstrating the tendency of availability bias [15]. The study from Lucas et al., identified availability bias as one of four main biases in clinical decision-making, but found it to have the lowest presence in comparison with premature closure, anchoring, and confirmation bias [16].

#### Confirmation bias

Confirmation bias is the tendency to seek information that confirms one’s beliefs and pay less attention to or being more critical to information presenting competing possibilities. For example, clinicians might overfocus on findings supporting their diagnosis, and under value information weakening their hypothesis [20]. Two studies report on confirmation bias [16, 17]. Fassier et al., suggest that when physicians make end-of-life decisions, they use physiological age as an argument to confirm their belief in a decision rather than to seek information to questions it [17]. Lucas et al., found in their registry study that confirmation bias was present in 55,6% of cases of care escalation to ICU in 2020 [16].

### Comprehension of current situation

#### Overconfidence bias

Overconfidence is the tendency to be more confident in one’s own abilities, presumptions, or predictions than objective reasonable. When exposed to overconfidence bias, people tend to act on incomplete information, making forecasts for patient outcomes with overconfidence in their own accuracy [14, 21].

Three studies reported on overconfidence bias [17, 22, 23]. Fassier et al., suggest in their study that physicians making decision alone might be more prone to overconfidence bias [17]. Another study conducted by Steinberg et al., found that providers are inaccurate when predicting survival and functional outcome after cardiac arrest in a third of their assessments, most errors are described to be optimistic [22]. A simulation study from Yang et al., presents that experienced critical care nurses did not show better calibration in their own confidence on accuracy compared to nursing students when identifying patients at risk of a critical event. They found that in general nursing students were underconfident and experienced critical care nurses were overconfident when compared to accuracy [23].

#### Premature closure

Premature closure is described as a tendency to accept a diagnosis without sufficient information. Basing judgement on characteristics that may be convincing but not decisive for a particular diagnosis, or by anchoring to early perceptions [14]. Three articles describe premature closure [16, 20, 24]. In the study from Lucas et al., they found premature closure to be the most prominent cognitive bias in their study of ICU upgrades due to non-concordant diagnosis [16]. Two other articles describe premature closure as making a clinical decision before having the necessary information to conclude [20, 24].

### Projection of future status

#### Omission bias

Omission bias is described as judging the potential harmful consequences of one’s own actions as worse than the same consequences of not doing anything. For example, a physician might be unsure about providing thrombolytic treatment to a stroke patient with mild symptoms and might incorrectly weigh the consequences of a potential bleed caused by treatment heavier than the consequences of not providing treatment [14, 20].

Three articles reported on omission bias [20, 25, 26]. Freshwater-Turner et al., presents a case report on a 15-year-old male where they reflect on the occurrence of omission bias. In their reflection they present that they judged the risk of bleeding from providing anticoagulation treatment worse than the risk of allowing clot propagation and pulmonary embolism [25]. Lighthall et al., discuss how clinicians in critical care typically are presented with decisions on “intervene or not”, they provide examples of *“deciding to minimize fluid administration and accepting declining renal function versus infusing fluids and risking an abdominal compartment syndrome”* [20]. Aberegg et al., presents that omission bias can lead to harmful inaction of not providing indicated treatment or unnecessary tests. They argue that when omission bias occurs, a degree of uncertainty is present [26].

### Contributing factors

Twelve of the included articles reported on contributing factors. Seven of twelve were primary research. Three were discussion articles and two were evidence synthesis. Following the inductive qualitative content analysis from Elo and Kyngäs [12], 17 condensed meaning codes were constructed and categorized into 3 categories that emerged.

### Lack of unbiased feedback

Three articles reported on lack of unbiased feedback. Lack of unbiased feedback refers to situations where the information used to evaluate decisions is distorted by the assumptions, actions, or systems that produced the decision. Steinberg et al., presents that healthcare providers making clinical decisions based on their predictions after cardiac arrest rarely receive unbiased feedback, and that this might result in poorly calibrated decisions [22]. Stanak et al., also describe how biased feedback contributes to development of self-fulfilling prophecies [19]. The study from Marsden et al., presents that during their interviews they found prehospital physicians to have limited awareness of biases affecting their decisions [15].

#### Social behaviour and beliefs

Five articles reported on social behaviour and beliefs [15, 19, 20, 26, 27]. Stanak et al., states that low survival rates for infants born at week 24 are affected by policies limiting treatment for these patients, and that this in turn will justify and validate the policy, contributing to social norms and institutional biases [19]. Ordaoobadi et al., presents that subtle cues such as grumbling and eye rolling from hospital personnel receiving geriatric trauma patients makes EMS clinicians question their decisions [27]. Aberegg et al., describes in their article how fear of regret, blame or legal consequences affects clinical decisions [26]. In the study from Marsden, they found that prehospital physicians had a lack of awareness of factors affecting their decision-making [15]. Lighthall et al., proposed that group consensus might promote conformity rather than protect against early closure or other biases [20].

#### Time pressure

Three articles reported on time pressure. Aberegg et al., reported that the time and effort required to change the status quo might be a contributing factor [26]. The article from Lucas et al., presents that patient overload may lead to increased use of cognitive short cuts, which may result in less analytic effort [16].

In the study from Yang et al. [23], they found that nurses became more confident in easy cases and less confident in difficult cases when under time pressure. Time pressure affects their motivation and confidence towards their favoured hypothesis, by a need for closure. This might contribute to the consideration of fewer hypotheses [23].

### Mitigating factors

Eleven of the sixteen included articles suggested mitigating factors. Five of eleven were primary research, 4 were discussion articles and 2 were evidence synthesis. Several factors were proposed; these are divided into three categories of mitigation factors.

#### Feedback and follow-up

Five of the included articles describe feedback and follow-up as a mitigating factor [19, 25, 28]. Three of the articles present the importance of creating a collective awareness of cognitive biases as a mitigating factor. They suggest that this is facilitated by providing unbiased feedback and follow-up [19, 25, 28].

Stanak et al., promotes that there is a need for clinicians to recognize biases [19]. Ordoobadi et al., states that if EMS clinicians received formal feedback and follow-up after handing-over geriatric trauma patients it would allow them to learn from each call [27]. Lighthall et al., presents that by providing feedback on cognitive performance, providers are able to build expertise from experience [20].

#### Organizational culture

Six of the included articles highlight organizational culture as an important topic in mitigating cognitive biases [16, 17, 20, 28–30]. The most cited organizational culture factor was the development of active group reflection [16, 20, 29]. Beldhuis et al., also states in their article that development of clinical decision support systems through the accumulating of data into actionable information for the clinician might contribute to mitigating cognitive biases in critical care [28]. In the article from Fiadjoe et al., they present that the implementation of checklists and a flat team structure encouraging all members to speak up and mitigate cognitive error cycles [30]. Fassier et al., state in their study that the use of shared decision-making and slowing down strategies might contribute to mitigation of cognitive biases [17].

#### Education and training

Seven articles present education and training as an important mitigation factor [16, 17, 25, 27–30]. Four articles highlight that debiasing should start with learning about clinical decision-making through educational programs, practicing decision-making, cognitive training such as metacognition, mindfulness, and creating awareness of one’s own thinking [16, 25, 28, 29]. Also, education and training should focus on specific biases regarding vulnerable patient groups, such as geriatric patients [17, 27]. Fiadjoe et al., presents that learning and practicing how to be receptive for input from all team members and to give feedback and input should be an aspect of education and training [30].

## Discussion

This scoping review sheds light on an important topic and has identified several cognitive biases that affect clinical decision-making in critical care that may be of great relevance to prehospital critical care. Furthermore, it identifies that the topic of cognitive biases and clinical decision-making in prehospital critical care has not been extensively explored, despite being acknowledged as an important contributor to adverse events and patient harm [5]. The identified literature in this scoping review reveals a growing body of literature surrounding cognitive biases and their mitigation in critical care decision-making that seems to be highly relevant for the prehospital practice and quality of patient care. The aim of this scoping review was to map current evidence and investigate, “What cognitive biases affects clinical decision-making in prehospital critical care”. A limited number of articles describing cognitive biases and clinical decision-making in critical care were identified, the findings were heterogenous, and none of the prehospital studies had cognitive biases as a main topic of research.

The description of the impact of cognitive biases and their contributing factors in the in-hospital critical care setting seems highly transferable to the prehospital setting. Most of the identified articles proposed contributing factors, these were categorized into lack of unbiased feedback, social behaviour and beliefs, as well as time pressure. In combination with the nature of high complexity and time pressure of clinical decision-making in prehospital critical care, the identified contributing factors suggest that prehospital clinicians may be particularly exposed to the potential impact of cognitive biases in their clinical decision-making. Prehospital services have been found to lack formal systems for providing unbiased feedback on clinical performance back to prehospital care providers [33–35]. The lack of unbiased feedback on clinical decision-making likely provides a challenging condition for the novice prehospital clinicians to build expert experience, and for experienced prehospital clinicians to adjust and refine their practice. When feedback is biased, it does not accurately reflect the true consequences of a decision, making it difficult for individuals or institutions to recognize errors, adjust behaviour, or improve future decisions. This limit learning and reinforces existing beliefs or practices, even if they are flawed. The identified prevalence of cognitive biases in clinical decision-making supports the mechanisms described by the heuristics and biases paradigm from Tversky and Kahneman [36]. Klein on the other hand researched how experienced decision makers make decisions in their operational natural setting. Klein claims that intuition grows from experience, and that skilled decision makers know that they can depend on their intuition [37]. The heuristics and biases paradigm from Tversky and Kahneman [36] have been seen in contrast to the Natural decision making (NDM) theory from Klein, and critiqued for having little interest in the value of expertise of decision-makers and the benefits of heuristics [38]. The theory of NDM provides a good foundation for understanding expert decision-making under uncertainty. If we were to view clinical decision-making purely by the concept of NDM, the heuristics and biases paradigm would claim that not taking biases into consideration is too simplistic. Kahneman and Klein state that “*a psychology that neglects predictable errors cannot be adequate*” [39]. Flin [40] presents that experts rely heavily on their situational awareness. This scoping review has proposed a categorization of cognitive biases after a deductive content analysis based on the levels of situation awareness model from Endsley [11].

The most reported cognitive biases were those that affected perception of elements in the current situation, such as anchoring bias, availability bias, confirmation bias and framing effect. Overconfidence bias, omission bias, status quo bias were also among reported. Even though these cognitive biases are important and found to affect clinical decisions, they might also represent the most well-known cognitive biases in healthcare. For instance, premature closure was found by Lucas et al. [16], to be the most prominent cognitive bias in their study, however they were the only primary research article that reported on premature closure. This is not to state that the most reported cognitive biases are more important than others as the prominence of specific biases likely indicate the lack of empirical studies on clinical decision-making and cognitive biases in these clinical settings.

Interestingly the most described contributing factors such as social behaviour and beliefs are presented to be related to biases such as loss aversion, representativeness, group thinking and availability. Time pressure as a contributing factor seems to be coherent with premature closure, triage cueing, overconfidence, and status quo. However, premature closure and triage cueing seem to be lacking research focus. The lack of unbiased feedback in prehospital services might affect both personal and institutional biases, one example is the article from Stanak et al. [19], presenting that, the guidelines that governs the treatment can lead us to justify them on the basis of the outcome. For instance, guidelines governing only comfort care for some traumatic brain injury patients, could lead us to believe that those patients are unsalvageable, due to low-survival rates we get from following the guidelines. Similar personal practice might be contributing to the development and occurrence of own personal biases.

Several mitigating factors that may reduce the risk of bias in decision-making were identified. Education and training were the most proposed category for mitigation factors, however, no evidence evaluating the impact of these mitigation factors were identified. Since biases operate at an unconscious level it is still unclear what effect education and training has on the occurrence of cognitive biases in clinical decision-making. However, together with the proposals of learning and practicing communication to give and receive feedback, it should be examined further. Providing unbiased feedback might also help clinicians to identify when they might be affected by cognitive biases, and in total provide clinicians the possibility to build expertise from experience. As both Kahneman and Klein agreed upon: *“The determination of whether intuitive judgments can be trusted requires an examination of the environment in which the judgment is made and of the opportunity that the judge has had to learn the regularities of that environment”* [39].

The main body of literature identified in this scoping review was published after 2015 with most articles published after 2019. This might indicate a growing acknowledgement and interest in this topic.

This scoping review has its strengths and limitations. First, it was conducted as the master thesis with only one reviewer performing the full-text screening, data extraction, data analysis, and the qualitative content analysis and categorization. This limitation is partly mitigated by the development and piloting of the search strategy in collaboration with a research librarian and a second non blinded review of titles and abstracts by the project supervisor.

## Conclusions

It is argued that critical care is one of the most decision intense fields of healthcare. This scoping review has mapped the current evidence and identified several cognitive biases that affect clinical decision-making and potentially contribute to errors that cause serious patient harm. According to the identified findings, leaders and decision makers should strive to establish systems to provide unbiased feedback on clinical performance for clinicians and facilitate awareness of cognitive biases in decision-making. Future research should focus on investigating cognitive biases in the field of prehospital critical care and specifically investigate biases that are less studied in critical care, such as premature closure, triage cueing, loss aversion, representativeness, and group thinking. Researchers should aim to establish how these cognitive biases affect clinical decisions in prehospital critical care, and what measures may mitigate the consequent errors, may reduce patient harm, and improve outcomes.

## Data Availability

No datasets were generated or analysed during the current study.
